# High Expression of Both Resistin and Fascin-1 Predicts a Poor Prognosis in Patients with Colorectal Cancer

**DOI:** 10.1155/2020/8753175

**Published:** 2020-04-26

**Authors:** Chao-Qun Wang, Yan Wang, Bi-Fei Huang, Chih-Hsin Tang, Zhang Du, Yue Zeng, Qian Wang, Jun-Kang Shao, Lu-Lu Jin

**Affiliations:** ^1^Department of Pathology, Affiliated Dongyang Hospital of Wenzhou Medical University, Dongyang, Zhejiang, China; ^2^Department of Medical Oncology, Affiliated Dongyang Hospital of Wenzhou Medical University, Dongyang, Zhejiang, China; ^3^Graduate Institute of Basic Medical Science, China Medical University, Taichung, Taiwan; ^4^Department of Pharmacology, School of Medicine, China Medical University, Taichung, Taiwan; ^5^Department of Biotechnology, College of Health Science, Asia University, Taichung, Taiwan; ^6^Department of Anus and Intestine Surgery, Affiliated Dongyang Hospital of Wenzhou Medical University, Dongyang, Zhejiang, China; ^7^Laboratory of Biomedicine, Affiliated Dongyang Hospital of Wenzhou Medical University, Dongyang, Zhejiang, China

## Abstract

Emerging evidence indicates that resistin and fascin-1 may possess a causal role in the development of several types of cancers. However, the clinical significance of resistin expression in colorectal cancer (CRC) tissues is unclear, and there are no reports of any correlation between resistin and fascin-1. Our analyses explored the expression of resistin in CRC tissue and analyzed the clinical and prognostic significance of the observed positive correlation between resistin and fascin-1. The rate of strongly positive resistin expression (27.5%) was significantly higher in CRC tissues than in normal colorectal tissues (5.2%). Strongly positive resistin expression is related to multiple poor prognostic factors in CRC, including depth of tumor invasion, lymph node metastasis, and tumor stage. In this study, survival was worse in CRC patients with high levels of both resistin and fascin-1 expression than in those with high levels of only one protein or normal levels of both proteins. We suggest that a combined high level of resistin and fascin-1 expression correlates reliably with survival in CRC, so it may serve as a potential therapeutic target.

## 1. Introduction

Colorectal cancer (CRC) is one of the most common types of cancers globally and is ranked amongst the top three malignancies in terms of morbidity and mortality [1, 2]. Resistin is a cytokine secreted by adipocytes that participates in the body's metabolism, inflammation, and autoimmunity through multiple molecular pathways [3]. While initial research focused on obesity and insulin resistance, resistin was later implicated in the occurrence and progression of various malignant tumors [4–9]. Evidence demonstrates that higher levels of circulating resistin increase the risk of developing CRC [10]. However, verification as to the expression of resistin in CRC tissue is limited to one report involving a small sample of CRC tissues [11]; the clinical significance has not been further clarified.

Overexpression of fascin-1, an actin-bundling protein, has been reported in several types of cancer [12–16]. In our previous study, we found that epidermal growth factor induced the expression of fascin-1 by activating p44/p42 MAPK (ERK1/2), which subsequently promoted breast cancer cell migration and invasion [17]. Other reports have shown that resistin promotes angiogenesis in osteosarcoma and proliferation of smooth muscle cells through p44/p42 MAPK (ERK1/2) signaling [9, 18]. Up until now, there have been no reports documenting an association between resistin and fascin-1.

In this study, we performed an immunohistochemical (IHC) analysis to detect resistin expression in CRC tissue samples obtained from a cohort of Chinese patients. We examined the association between levels of resistin and fascin-1 expression and sought to clarify the clinicopathologic and prognostic significance of this association.

## 2. Materials and Methods

### 2.1. Patients and Tissue Samples

CRC tissue samples were obtained from 360 untreated Chinese patients who were undergoing primary surgical treatment at the Affiliated Dongyang Hospital of Wenzhou Medical University (Dongyang, Zhejiang, China) between 2008 and 2015. Seventy-seven samples of adjacent normal colorectal tissue were also obtained following surgical resection. Clinicopathologic characteristics were determined for all patients based on their medical records. Follow-up information was available for 271 patients with CRC; the median follow-up time was 61 months (range, 6–75 months). The Ethics Committee of the Affiliated Dongyang Hospital of Wenzhou Medical University approved this study, and written informed consent forms were signed by all patients or their guardians. All study methods satisfied the relevant guidelines and regulations issued by the Affiliated Dongyang Hospital of Wenzhou Medical University.

### 2.2. Tissue Array Preparation

The Quick-Ray® UT-06 (Unitma Co., Ltd., Seoul, Korea) tissue microarray system was used to prepare tissue specimens, and we used the Quick-Ray premade recipient block (UB-06) wax model. Three representative sites from each CRC tissue were selected for sampling, and a tissue array with a diameter of 1 mm was made following the manufacturer's protocol.

### 2.3. IHC Analysis

IHC staining of paraffin-embedded tissue array sections was conducted using the Envision System (Dako, Glostrup, Denmark), as described previously [19]. The primary antibodies used included anti-resistin mouse monoclonal antibody (clone C-10, diluted at 1 : 25; Santa Cruz Biotechnology, Santa Cruz, USA) and anti-fascin-1 mouse monoclonal antibody (clone 55k-2, diluted at 1 : 100; Santa Cruz Biotechnology).

### 2.4. Assessment of Staining

The entire tissue array section was scanned and scored separately by 2 pathologists. Staining intensity was scored on a 4-point scale from 0 (negative) to 1 (weak), 2 (moderate), or 3 (strong). Staining extent was scored on a 5-point scale from 0 (0%) to 1 (1%–25%), 2 (26%–50%), 3 (51%–75%), or 4 (76%–100%). A sum of ≥3 for staining intensity and extent scores and percentage of >5% for invasiveness of tumor cells with unequivocal cytoplasmic staining were considered to be positive for resistin. A combined sum of ≥6 for the scores was considered to be strongly positive for resistin [19]. The staining intensity and extent of fascin-1 expression were also recorded in CRC cancer cells, using the same scoring criteria as described previously [19]. High levels of resistin and fascin-1 expression are expressed as strongly resistin-positive and fascin-1-positive, respectively.

### 2.5. Patient Follow-Up

Each patient was followed-up postoperatively by telephone call and thereafter at 6-monthly hospital appointments; follow-up was discontinued in the event of patient death. A diagnosis of local CRC recurrence was made by clinical or histology results. Relapse-free survival (RFS) was defined as the time from surgery to relapse/metastasis; overall survival (OS) was the time from surgery to death (excluding nontumor-related deaths).

### 2.6. Statistical Analysis

Statistical analyses were conducted using SPSS version 19.0 (SPSS Inc., Chicago, IL, USA). Differences between groups were compared using Pearson's chi-square test for qualitative variables. The correlation between resistin and fascin-1 protein expression was assessed by Spearman's correlation analysis. Patient RFS and OS rates were analyzed using the Kaplan-Meier method and compared using log-rank analysis. *P* < 0.05 was considered to be statistically significant.

## 3. Results

### 3.1. Expression of Resistin in Colorectal Tissue and Its Relationship with the Clinicopathologic Characteristics of Patients

**Figure 1:**
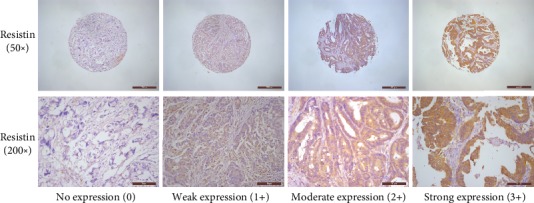
Resistin expression levels in colorectal cancer patients. Colorectal cancer tissue specimens were immune-stained with anti-resistin antibody, photographed using an optical microscope, and scored from 0–3 for staining intensity of resistin expression.

The rate of positive resistin expression in CRC tissue specimens was 93.3% (336/360), which included 99 (27.5%) strongly positive cases, while the rates of positive and strongly positive resistin expression in normal colorectal tissues were 96.1% (74/77) and 5.2% (4/77), respectively (Figure 1). The rate of strongly positive resistin expression was significantly higher in CRC tissues than in normal colorectal tissues (*P* < 0.01) (Table 1). We also observed significant associations between a strongly positive level of resistin expression and clinical parameters in CRC tissue specimens, including tumor stage (*P* = 0.022) and lymph node metastasis (*P* = 0.009) (Table 2). In regard to the depth of invasion, the rate of strongly positive resistin expression was higher in the T3/T4 group (29.4%, 88/299) compared with the Tis+T1/T2 group (18.0%, 11/61), but the between-group difference was not statistically significant (*P* = 0.069).

### 3.2. Correlation of Resistin and Fascin-1 Expression in CRC

**Figure 2:**
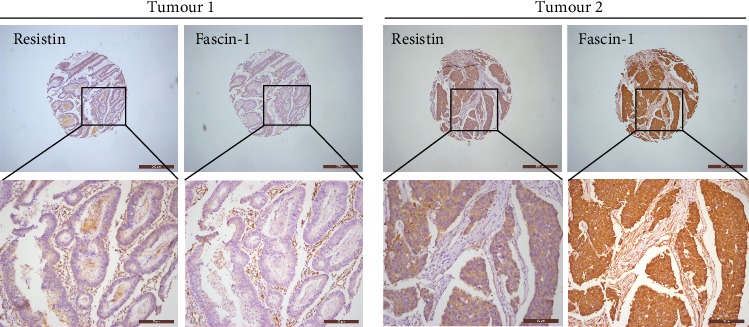
A tendency of positive protein levels between resistin and fascin-1 in colorectal cancer: human colorectal cancer tissue microarrays were immune-stained with anti-resistin and anti-fascin-1 antibodies. Representative staining pictures of tumors are shown.

IHC analyses detected fascin-1 expression in 233 CRC cases. The rate of positive fascin-1 expression in CRC tissue specimens was 38.2% (89/233). When we analyzed the relationship between resistin and fascin-1 expression, we found a significantly higher level of fascin-1 expression in CRC tissue specimens from cases that were strongly positive for resistin (50.0%, 27/54) compared with those that were not strongly resistin-positive (34.6%, 62/179; *P* < 0.05, Table 3) (Figure 2). Spearman correlation analysis revealed a significantly positive correlation between strongly positive levels of resistin expression and fascin-1 positive expression in CRC tissue specimens (*R* = 0.133, *P* = 0.042).

### 3.3. High Expression of Both Resistin and Fascin-1 Is Associated with Worse Survival in CRC

**Figure 3:**
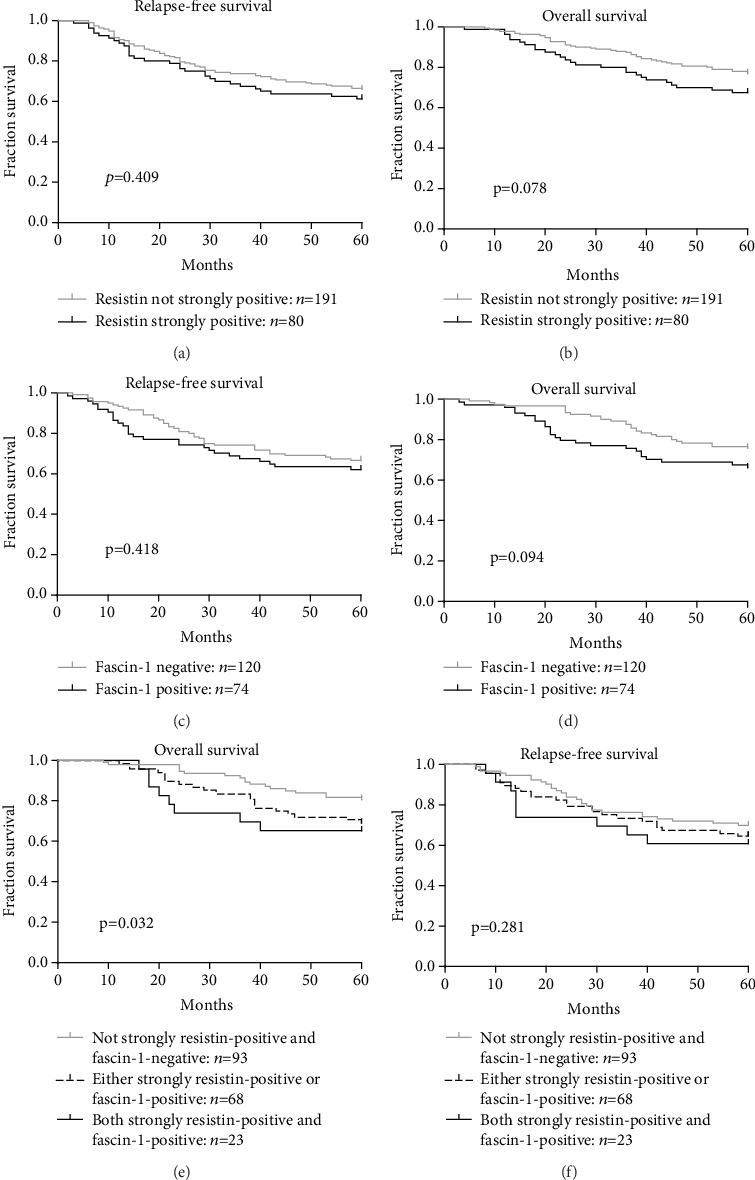
Resistin expression was associated with the survival of patients with colorectal cancer. (a, b). The associations of resistin expression with relapse-free survival (RFS) (a) and overall survival (OS) (b) were analyzed. (c, d). The associations of fascin-1 expression with RFS (c) and OS (d) were analyzed. (e, f). Kaplan-Meier curves for OS (e) and RFS (f) of combined high expression of resistin and fascin-1 in colorectal cancer. *P* values were calculated using the Mantel-Cox log-rank test.

To assess the potential impact of high levels of expression of both resistin and fascin-1 on patient survival, we analyzed resistin and fascin-1 expression in relation to RFS and OS rates in patients with CRC. As shown in Figures 3(a) and 3(b), CRC patients who were strongly resistin-positive had worse RFS and OS than those who were not strongly resistin-positive, although the data did not support an unequivocal association (*P* = 0.409 and *P* = 0.078, respectively). Similarly, as shown in Figures 3(c) and 3(d), fascin-1-positive patients experienced worse RFS and OS, but there was no evidence of an unequivocal association (*P* = 0.418 and *P* = 0.094, respectively).

In further analysis of the potential impact of high levels of both resistin and fascin-1 expression on patient survival, as shown in Figures 3(e) and 3(f), patients whose primary tumors were both strongly resistin-positive and fascin-1-positive (*n* = 23) had a mean OS of 47.5 months (an estimated 5-year OS rate of 65.2%), while patients whose tumors were either strongly resistin-positive or fascin-1-positive (*n* = 68) had a mean OS of 51.4 months (an estimated 5-year OS rate of 69.1%), whereas patients whose tumors were not strongly resistin-positive and fascin-1-negative (*n* = 93) had a mean OS of 55.2 months (an estimated 5-year OS rate of 80.6%, *P* = 0.032; Figure 3(e)). Patients whose tumors expressed high levels of both resistin and fascin-1 experienced worse RFS, although evidence of an unequivocal association was lacking (*P* = 0.281, Figure 3(f)).

## 4. Discussion

The adipokine resistin participates in several physiologic and pathologic processes throughout the body, including metabolism, inflammation, autoimmunity, and various cancers, including CRC [3–9]. Previous research has documented an association between higher levels of circulating resistin and an increased risk for CRC [10]. However, the clinical significance of resistin expression in CRC tissue specimens is unclear. In our study, we observed a significantly higher rate of strongly positive resistin expression in specimens from CRC tissue, compared with that from normal colorectal tissue. Strongly positive resistin expression is significantly associated with a number of clinical parameters in CRC patients, including tumor stage and lymph node metastasis. These results suggest that high levels of resistin expression in CRC tissues may be linked to disease progression.

Fascin-1, an actin-bundling protein, is normally expressed in endothelial, mesenchymal, and neuronal cells and is low or absent in normal epithelial cells. Overexpression of fascin-1 has been reported in several types of cancers, including the lung, colon, stomach, ovary, and breast [12–16]. Our previous study found that epidermal growth factor induced the expression of fascin-1 via activation of the p44/p42 MAPK (ERK1/2) pathway, which subsequently promoted breast cancer cell migration and invasion [17], while other reports have shown that resistin also promotes osteosarcoma angiogenesis and the proliferation of smooth muscle cells through the p44/p42 MAPK (ERK1/2) pathway [9, 18]. However, whether any association exists between resistin and fascin-1 expression has been unclear. We therefore sought to determine the clinical prognostic significance of our observed highly significant correlation between high levels of resistin and fascin-1 expression in CRC tissue. We hypothesized that fascin-1 utilizes the resistin-MAPK signaling pathway to play functionality.

Survival analysis shows that both resistin and fascin-1 impact adversely upon survival in CRC, but neither protein alone has a significant impact. CRC patients who expressed high levels of both resistin and fascin-1 expression had worse OS than all other CRC patients. These results suggested that resistin and fascin-1 are potential clinical biomarkers of disease progression and prognosis in CRC.

Identification of therapeutic targets that act synergistically has the potential to improve survival and quality of life in patients with malignant tumors. Our data indicate that cotargeting resistin and fascin-1 is a useful therapeutic strategy in CRC. Further investigations are warranted to elucidate the molecular mechanisms that explain how resistin regulates fascin-1 expression in CRC.

## Figures and Tables

**Table 1:** Strongly positive resistin expression in colorectal tissue specimens.

Group	No.	Resistin expression	
		Not strongly positive, *n* (%)	Strongly positive, *n* (%)
Normal colorectal	77	73 (94.8%)	4 (5.2%)
Colorectal cancer	360	261 (72.5%)	99 (27.5%)^∗^

^∗^ *P* < 0.01.

**Table 2:** Association of strongly positive resistin expression with clinical pathologic parameters in patients with colorectal cancer.

Variables	No.	Strongly positive resistin expression, *n* (%)	*P* value
Gender			
Male	215	57 (26.5%)	0.609
Female	145	42 (29.0%)	
Age (years)			
<60	102	30 (29.4%)	0.609
≥60	258	69 (26.7%)	
Tumor site			
Right colon	87	27 (31.0%)	0.622
Left colon	82	20 (24.4%)	
Rectum	191	52 (27.2%)	
Tumor grade			
High	36	12 (33.3%)	0.686
Medium	288	78 (27.1%)	
Low	36	9 (25.0%)	
Depth of invasion			
Tis+T1/T2	61	11 (18.0%)	0.069
T3/T4	299	88 (29.4%)	
Lymph node metastases			
-	200	44 (22.0%)	0.009
+	160	55 (34.4%)	
Tumor stage			
I	42	5 (11.9%)	0.022
II	151	37 (24.5%)	
III	141	49 (34.8%)	
IV	26	8 (30.8%)	

**Table 3:** The relationship between levels of resistin and fascin-1 expression in patients with colorectal cancer.

Group	No.	Fascin-1 expression	
		Negative, *n* (%)	Positive, *n* (%)
Resistin not strongly positive	179	117 (65.4%)	62 (34.6%)
Resistin strongly positive	54	27 (50.0%)	27 (50.0%)^∗^

^∗^ *P* < 0.05.

## Data Availability

All data generated or analyzed during this study are included in this published article.

## Abbreviations

IHC: Immunohistochemistry
MAPK: Mitogen-activated protein kinase
RFS: Relapse-free survival
OS: Overall survival
ERK1/2: Extracellular signal-regulated kinase 1/2.

## Additional Points

*Novelty and Impact*. Currently, the expression of adipokine resistin in colorectal cancer tissue and its clinical significance remains unclear. Our study has identified high levels of resistin expression in colorectal cancer tissue and a positive correlation between this expression and disease progression in patients. In particular, survival was worse in those patients with high levels of both resistin and fascin-1 expression than in those with high levels of only one protein or normal levels of both proteins. These findings indicate that resistin and fascin-1 are appropriate clinical biomarkers for colorectal cancer and that simultaneously targeting these proteins is a potentially useful therapeutic strategy in this disease.
